# New Orders to an Old Soldier: Optimizing NK Cells for Adoptive Immunotherapy in Hematology

**DOI:** 10.3390/biomedicines9091201

**Published:** 2021-09-11

**Authors:** Mehmet Gunduz, Pinar Ataca Atilla, Erden Atilla

**Affiliations:** 1 Department of Hematology, Biruni University, Istanbul 34010, Turkey; drmgunduz02@gmail.com; 2 Interdisciplinary Stem Cells and Regenerative Medicine Ph.D Program, Stem Cell Institute, Ankara University, Ankara 06520, Turkey; drpinarataca@gmail.com; 3 Department of Hematology, Mersin State Hospital, Korukent District, 96015 St., Toroslar 33240, Turkey

**Keywords:** NK cells, CAR NK cells, adoptive immunotherapy

## Abstract

NK (Natural Killer) cell-mediated adoptive immunotherapy has gained attention in hematology due to the progressing knowledge of NK cell receptor structure, biology and function. Today, challenges related to NK cell expansion and persistence in vivo as well as low cytotoxicity have been mostly overcome by pioneering trials that focused on harnessing NK cell functions. Recent technological advancements in gene delivery, gene editing and chimeric antigen receptors (CARs) have made it possible to generate genetically modified NK cells that enhance the anti-tumor efficacy and represent suitable “off-the-shelf” products with fewer side effects. In this review, we highlight recent advances in NK cell biology along with current approaches for potentiating NK cell proliferation and activity, redirecting NK cells using CARs and optimizing the procedure to manufacture clinical-grade NK and CAR NK cells for adoptive immunotherapy.

## 1. Introduction

Immunobiology and immunotherapy of hematological malignancies have captured great interest in recent years. NK (Natural Killer) cells are components of the innate system that identify and kill tumor- and virus-infected cells in a major histocompatibility complex (MHC) unrestricted fashion. Unlike T cells, which recognize through an antigen-specific T-cell receptor (TCR) and express receptors encoded by rearranging genes, NK cells have activating and inhibitory receptors (killer immunoglobulin receptors, or KIRs) that ligate MHC molecules [1,2,3]. Tumor cells down-regulate or lose the MHC class I expression and become susceptible to lysis by NK cells. Several activating NK cell receptors and co-stimulatory molecules recognize tumors [4]. NK cells also exhibit antigen-dependent cellular cytotoxicity (ADCC) by detecting antibodies on tumor cells through the low-affinity Fcγ CD16 receptor [5].

NK cells have become an attractive modality in adoptive immunotherapy during the last two decades due to the growing research about NK cell biology that has elucidated the insufficient anti-tumor effect and expansion. Initially, trials examined the ex vivo activated and expanded primary peripheral blood (PB) NK cells or NK cell lines (e.g., NK-92) in autologous and allogeneic settings [1,2]. Umbilical cord blood (UCB)-derived NK cells are demonstrated to be younger, recover better after cryopreservation and have stronger proliferation potential. Manufacturing NK cell-based immunotherapies from induced pluripotent stem cells (iPSCs) has prevented long production times while maintaining “off-the-shelf” capabilities [6].

NK-cell mediated antitumor immunotherapy can be enhanced by checkpoint blockade, bi- and tri-specific killer engagers (BIKEs and TriKEs), anti-KIR monoclonal antibodies and chimeric antigen receptor (CAR)-engineered NK cells (CAR-NK cells). Cytokines play essential roles in NK cell expansion and potentiating NK cell therapy products [7,8]. Genetic modifications have further improved the specificity, strength and efficacy of NK cell-based immunotherapies. Today, the optimal time for NK cell infusions has not been determined. Non-modified or modified NK cells can be used as maintenance therapy after chemotherapy or can be combined with autologous or allogeneic stem cell transplantation. In this paper, we will review the mounting evidence for adoptive NK cell therapy in hematology: NK cell biology, autologous and allogeneic applications of NK cell therapies, the principles of CAR-NK cell therapies as well as challenges and solutions for the manufacturing process. 

## 2. Recent Advances in NK Cell Biology

NK cells are elements of innate defense and have been re-classified as cytotoxic innate lymphoid cells (ILCs) that show cytolytic activity, produce pro-inflammatory response and express Eomesodermin (Eomes) and T-box transcription [9,10]. NK cells are educated for “auto-reactivity” and compose at least one inhibitory receptor for self HLA class I antigens. In addition to HLA-specific inhibitory and activating NK receptors—Killer-cell immunoglobulin-like receptors (KIRs), NK cells express inhibitory receptors including PD-1, TIGIT, CD96, TIM-3 and CD161 for anti-tumor activity [11]. NK cell development was thought to occur only in bone marrow, but recent evidence shows that NK cells also mature in secondary lymphoid organs (including the tonsils, lymph nodes, thymus and liver) [3]. During the NK cell differentiation process, the surface receptors CD161, 2B4 and NKp80 were detected in different stages; CD94/NKG2A is expressed first while KIRs appear only at later stages [12].

NK cells have historically been considered as “naturally” cytotoxic cells with limited life span and proliferative capacity, but recent research indicates that NK cells also require priming of various factors such as IL-15, IL-2, IL-12 or IL-18 for maximum effector function [13]. Early clinical trials showed that administration of exogenous IL-2 facilitated NK cell expansion and persistence [14]. IL-15 plays a role in NK cell development and promotes NK cell survival through expression of anti-apoptotic factor Bcl-2 [15]. Miller and colleagues showed that IL-15 has superior activity to IL-2 for in vivo NK cell persistence [16].

NK cells not only function in innate immunity but also obtain immunological memory like T and B cells in adaptive immunity. Memory-like NK cells develop following infection with, for example, human cytomegalovirus (CMV) and respond to a cytokine cocktail (IL-12, IL-15 and IL-18) [17]. The memory-like response was correlated with the expression of CD94, NKG2A and CD69 and a lack of CD57 and KIR in CD56-dim NK cells [18]. When NK cells are stimulated with cytokines, immunomodulator-semaphoring 7A (SEMA 7A) is upregulated on NK cells, maintaining increased functionality [19].

## 3. Impaired NK Cell Function in Hematological Malignancies

In hematological malignancies, impaired function by chemotherapy, radiation and immunosuppressants and decreased numbers of NK cells have been demonstrated in many studies [20]. In acute myeloid leukemia (AML), patients with a defective NK cell profile, classified by distinct transcriptional modifications in pathways involved in cytotoxicity, intracellular signaling and metabolism, had higher risk of relapse [21]. AML patients demonstrate low expression of major activating NK receptors, increased expression of CD94/NKG2A inhibitory receptors, impaired NKG2D-mediated NK cell activity and low production of TNF-α and IFN-® [22]. The DNAM-1 receptor/ligand axis is altered in patients with AML, causing poor NK cell conjugation and killing [23]. AML blasts activate the aryl hydrocarbon receptor pathway, which induces miR-29b expression, impairing NK cell precursors’ maturation and functions [24]. Similarly, exosomes from leukemia/lymphoma cells can express NKG2D-L, which down-regulate the surface NKG2D expression and inhibit NK cell activation [25]. In chronic lymphocytic leukemia (CLL), soluble BAG6 (NKp30-ligand) competes for induction of NK cell activation via NKp30 [26]. Manso and colleagues defined NK cell reduction, as well as abnormally up-regulated genes (HIF-1α, GATA-1, PU.1 and GATA-2), as the basis of the immunodeficiency in CLL [27].

NK cell dysfunction is present in the tumor microenvironment. NK cell cytolytic function is impaired by hypoxia because of the decreased surface expression of activating receptors such as NKp46, NKp30, NKG2D and CD16 as well as overexpression of adenosine nucleotidase, CD73 [28,29]. Hypoxia can up-regulate checkpoint molecules such as PD-L1 and down-regulate the NKG2D ligand MICA on tumor cells [30]. In hematological malignancies, Galactin-9 secreted by blasts and high levels of PD-1 in circulating and intra-tumoral NK cells impair the cytolytic function of NK cells [31,32].

## 4. The Role of NK Cell Therapy in Hematological Malignancies

### 4.1. Administration of Autologous NK Cells

The initial study administering autologous IL-2-activated NK cell-rich populations or intravenous IL-2 infusions in lymphoma patients did not produce a significant effect compared with controls [33]; see Table 1. Several approaches have augmented the antibody-dependent cellular cytotoxicity (ADCC) of autologous NK cell therapy: inserting anti-tumor monoclonal antibody, checkpoint receptor blockers, bi- and tri-specific killer engagers (BiKEs and TriKEs) and cytokine-induced memory NK cells.

Deng et al. reported that when the anti-CD20 antibody rituximab was combined with NK cells, increased cytokine release (IFN-γ and TNF-α) and cytotoxicity were observed in vitro [48]. Additionally, rituximab induced NK-mediated ADCC when NK cells were expanded with K562-mbIL15-41BBL cells and IL-2 [49]. In a mouse model, the combination of rituximab and anti-KIR monoclonal antibody enhanced the anti-tumor effect of NK-cell mediated killing [50]. A phase I study conducted by ex vivo expanded autologous NK cell therapy combined with rituximab-containing chemotherapy showed that seven of nine lymphoma patients maintained complete response with a median duration of 44 months (range, 6–56 months); higher levels of stimulatory NK cell receptors were detected with response [34]. 

An anti-KIR monoclonal antibody (IPH2101-lirilumab) targeting KIR2DL1, KIR2DL2 and KIR2DL3 to block the interaction of inhibitory receptors on the surface of NK cells with their cognate HLA class I ligand limited side effects in AML and myeloma, but the anti-tumor efficacy was also limited [51,52]. Conversely, a monoclonal antibody targeting KIR3DL2, IPH4102 is promising in patients with cutaneous T-cell lymphoma, predominantly with Sezary syndrome [53]. There are ongoing trials hypothesized to induce an anti-tumor effect when anti-KIR antibody is administered in combination with anti-PD-1, anti-SLAM7, anti-CTLA-4, 5-azacytidine or daratumumab [1,2]. Daratumumab, a monoclonal antibody against CD38, is a feasible option when NK cells have CD38 knocked out by CRISPR/Cas9 to prevent fratricide [54]. Checkpoint receptor blockage through PD-1 or PD-L1 activated an NK response in mouse models of several cancers, including lymphoma. Activated NK cells express PD-1, interact with PD-L1+ tumor cells and down-regulate NK cell-mediated immunity. Other checkpoint inhibitors against CD96, TIGIT or TIM-3 enhance anti-tumor activity in various solid tumors [12].

BiKEs and TriKEs have been manufactured with CD16scFv, which can trigger NK cell function much more efficiently than natural CD16-Fv interaction to create a potent immunological synapse between NK cells and tumor cells [12]. Gleason et al. reported that a CD16 × CD33 BiKE induced NK cell function and enhanced degranulation against myelodysplastic syndrome (MDS) and CD33+ myeloid-derived suppressor cells (MDSC) [55]. The 161519 TriKE was designed to bind CD16 in NK cells as well as CD19 in chronic lymphocytic leukemia (CLL) cells and induces NK cell expansion and killing via IL-15 [56]. Sarhan et al. demonstrated that MDS NK cells showed more enhanced function and expansion with 161533 TriKE than with 1633 BiKE [57].

### 4.2. Administration of Allogeneic NK Cells

Although autologous NK cell therapies have useful effects, the aggressiveness of hematological malignancies, tumor escape and manufacturing failures because of the low number and compromised function of patient-derived NK cells have prompted interest in allogeneic NK cells for an “off-the-shelf” approach. Indeed, this process requires depleting T cells and/or regulatory T cells from the product to prevent graft versus host disease (GVHD) or lympho-proliferative disorders [58]. 

A pioneering study with successful allogeneic adoptive transfer of NK cells from a HLA-haploidentical donor in AML, demonstrated by Miller et al., confirmed the notion that KIR mismatch with tumor MHC may lead to greater cytotoxicity [14]. Complete hemtologic remission was achieved in 5 of 19 in poor-prognosis patients with AML under intensive cyclophosphamide and fludarabine conditioning regimens. In 10 pediatric patients, complete remissions (CRs) were achieved by KIR ligand-mismatched CD3-depleted and CD56-enriched NK cells (median dose, 26 × 10^6^/kg) and six doses of IL-2 (1 million U/m^2^) without graft versus host disease (GVHD) and remained in CR for 2 years [37]. In 57 refractory AML patients, the expansion of haploidentical NK cells was greater in 15 patients that received host regulatory T cell-depleted IL-2 diphtheria fusion protein (IL2DT) following cyclophosphamide and fludarabine than in patients who did not receive IL2DT (27% vs. 10%). The CR rate at day 28 was improved in patients with IL2DT (%53 vs. %21, *p* = 0.002) [59]. Bachanova et al. reported six patients with advanced B-cell non-Hodgkin lymphoma that received rituximab, cyclophosphamide and fludarabine followed by CD3-depleted NK cell-enriched cell products followed by subcutaneous IL-2 (10 × 10^6^ units/6 doses). The treatment did not cause major toxicity, and four of six patients showed a clinical response at 2 months. However, the inadequate immunodepletion and host Treg population affects NK cell survival and expansion unfavorably [40]. 

Transfusing haploidentical, T-cell depleted, KIR-ligand mismatched NK cells after conditioning therapy with melphalan and fludarabine in advanced multiple myeloma following autologous stem cell transplantation caused no significant toxicity; further blocking of inhibitory KIR ligands with anti-human leucocyte antigen antibody enhanced killing of multiple myeloma cells [60]. Lymphodepletion with busulfan, fludarabine and ATG followed by IL-2 activated haploidentical NK cells showed increased efficacy with delivery of CD56+ cells (*p* = 0.022) in high-risk AML, MDS and CML without an increase of GVHD [61]. NK cells isolated from haploidentical donors and activated with CTV-1 leukemia cell line lysate in a phase I trial showed a prolonged relapse-free survival (RFS) period in high dose of infusion (337 days, 3 × 10^6^) [62]. Recombinant human IL-15 also induced NK cell expansion and haploidentical transfer-induced remission in 35% of AML patients [42]. Another approach to maximize the anti-leukemia potential of NK cells is to pre-activate NK cells with IL-12, IL-18 and IL-15 to differentiate them into cytokine-induced memory-like NK cells. In a phase I trial using adoptively transferred cytokine-induced memory-like NK cells in AML, four of nine patients achieved CR [35]. 

The first-in-human study of NK cell products generated from CD34+ hematopoietic stem and progenitor cells (HSPC) of partially HLA-matched UCB units demonstrated that UCB-derived NK cells were well tolerated without a significant toxicity, and two of four patients with minimal residual disease (MRD) before infusion became MRD negative for 6 months [46]. From twelve multiple myeloma cases, UCB-derived NK cells were administered for MM patients undergoing high dose chemotherapy and autologous hematopoietic stem cell transplantation (auto-HCT), and 10 patients achieved at least very good partial responses [47]. 

In May 2021, the encouraging interim data from the phase 1 trials of “off-the-shelf” iPSC-derived NK cells against relapsed/refractory AML were released. In the dose escalation study of FT516, an iPSC line engineered to express a non-cleavable CD16 (hnCD16) Fc receptor, six patients had a reduction in the bone marrow blasts from nine patients. Complete remission with incomplete hematologic recovery (CRi) was detected in three patients without dose-limiting toxicities in any of the patients [44]. The following month, successful results were announced from the B-cell NHL cohort. Eight of eleven patients had objective responses, including six patients who achieved complete response [45]. The same group modified the clonal master iPSC cell line FT516 to FT538 in order to prevent its down-regulation and enhance its binding to tumor-targeting antibodies, along with adding an IL-15 receptor fusion (IL-15RF) and deleting the CD38 gene (CD38KO). From three AML patients, one achieved CRi [44]. FT596 was engineered with three anti-tumor functional modalities: chimeric antigen receptor that targets CD19, non-cleavable CD16 Fc receptor and IL-15RF. From 14 patients, 10 achieved objective response with 7 CR [45]. Table 1 summarizes the major clinical trials with allogeneic NK-cell therapy. 

### 4.3. NK Cell Engineering—“CAR-NK Cell Therapy”

Despite the tremendous efforts and considerable progress that has been achieved in adoptive NK-cell immunotherapy, a certain number of tumor cells with genetic or epigenetic variations can still bypass immunological surveillance [63]. To overcome the inhibition of the immune response and tumor escape, genetic modulation of NK cell-associated receptor expression is promising. A CAR is a genetically engineered protein composed of three parts: an extracellular domain derived from a single-chain fragment (scFv) targeted to a special antigen with high affinity binding, a transmembrane domain and an intracellular signaling domain. CAR technology was first applied to T cells, generating CAR-T cells, but some drawbacks have been demonstrated. Major limitations include the high risk of graft versus host disease (GVHD) in allogeneic use, high manufacturing costs and adverse events such as cytokine release syndrome (CRS) or neurotoxicity [64,65,66]. Conversely, CAR-NK cells do not cause GVHD and can be obtained from healthy third-party donors, making them suitable for “off-the-shelf” use. Adverse events have been observed less frequently; activated NK cells produce useful and safe cytokines such as IFN-γ and GM-CSF [67]. CAR-NK cells can also produce cytotoxic effects by using their native activating NK receptors, especially when tumor cells lose the antigen expression targeted by CARs, so the cytotoxic function of CAR-NK cells is not CAR-restricted [68]. On-target/off-tumor effects occur rarely because of the short life of NK cells [69].

Shimasaki et al. reported the considerable cytotoxicity in in vitro and in vivo models of B-cell leukemia of CAR-NK cells derived from peripheral blood after electroporation with the corresponding mRNA (anti-CD19-BB- ζ) [70]. For B-cell lymphoma and leukemia, Oelsner et al. engineered NK-92 cell line cells by lentiviral gene transfer to express CD19 CARs (CD3 ζ, CD28-CD3 ζ or CD137-CD3 ζ). Although less activity was observed with CD137-CD3 ζ co-stimulation, all CAR NK-92 cells retained cytotoxicity in vitro and in a Raji xenograft model in vivo [71]. UCB-derived NK cells transduced with a retroviral vector incorporating genes for CAR-CD19, IL-15 and inducible caspase-9-based suicide gene (iC9) killed CD19 positive cell lines and prolonged survival in a Raji xenograft lymphoma murine model [72]. CAR-NK cells against CD20, CD138 and CS-1 showed promising results in B-cell malignancies and multiple myeloma CD5 in T-cell malignancies in preclinical studies (Table 2). To our knowledge, different activation signals in CAR-NK cells have been compared in only solid tumors [73].

Not many clinical trials of CAR-NK cells against hematological malignancies are listed in clinicaltrials.gov (Table 3). Tang et al. safely administered the first CD33-CAR-NK-92 cells against relapse refractory acute myeloid leukemia (AML) in three patients. Patients had mild fever and cytokine release, but the response was transient (NCT02944162) [81]. HLA-mismatched anti-CD19 CAR-NK cells derived from cord blood with IL-15 and iCas9 were expanded on K562-mbIL21 and 4-1BB ligand feeder cells and administered to 11 relapse refractory B-cell lymphoma patients. The treatment was tolerated without major toxic effects or cytokine release syndrome. Among the 11 patients, 8 (73%) had a response, 7 had complete remission and 1 had remission [82] (NCT03056339).

## 5. Challenges in Manufacturing of Clinical-Grade NK and CAR-NK Cell Therapies and Strategies to Overcome Them

The major obstacles that have hindered the clinical application of NK cells are their low expansion profile and viability. Although not precisely stated, a meaningful response has been established in clinical trials when large numbers of NK cells, on the order of 10^5^–10^8^ NK cells/kg, were infused. Improved NK cell maturation and expansion ex vivo have been achieved with cytokines alone (IL-2, IL-12, IL-15, IL-18 and/or IL-21) or in combination with co-stimulatory antibodies/agonists, feeder cells generated from autologous mononuclear cells, EBV-transformed lymphoblastoid cell lines (EBV-LCLs) or tumor cells (K562)/feeder cells that have been genetically modified to express membrane-bound cytokines and co-stimulatory molecules such as 4-1BB, MICA, IL15 and IL21 [83,84,85,86,87]. Clinical products are only released without the presence of K562 cells or transformed LCLs [88]. Administering pre-infusion lympho-depleting therapy (e.g., cyclophosphamide and fludarabine) and repeated NK cell infusions after adoptive transfer also improved the expansion profile [21]. Some groups demonstrated that administering hematopoietic growth factors that stimulate erythropoietic receptor (EPOR) or thrombopoietin receptor (c-MPL) were safer than exogenous cytokines to increase NK cell survival and cytotoxicity [89]. Sutlu et al. stimulated 9.8 × 10^9^ NK cells derived from peripheral blood in a closed, automated, sterile, large-scale bioreactor consisting of a bag on a heated rocking or static platform under feeder-free GMP conditions after 21 days [90]. Miltenly’s Prodigy, Lonza’s Wave and G-Rex static bioreactors are automated and closed expansion equipments that have facilitated the manufacture of GMP-grade products [5]. Although large-scale generation of GMP-grade products is possible using serum-free commercial media such as AIMV media (Life Technologies, USA), X-VIVO media (BioWhittaker, Belgium) and SCGM media (CellGenix, Germany) [91], there are no standard manufacturing techniques because of the variation of culture conditions throughout clinical trials. In an optimization study of GMP-compliant manufacturing methods in CliniMACS Prodigy, NK MACS and TexMACs media achieved the highest NK cell purity; the highest fold of expansion was achieved by K562mbIL21-41BBL cells [92].

Similar problems appear in CAR-NK cell therapies. To solve this issue, NK cells can be co-cultured with leukemia cell line K562 as feeder cells transduced with membrane-bound cytokines (IL-15 or IL-21) [93]. Recently, next-generation CAR-T cell enhancements have been translated into CAR-NK cell approaches. When IL-15 was integrated with CD19 CAR-NK cells against lymphoma, the cells expanded in vivo and remained detectable for at least a year after infusion [82]. In a preclinical study, Wang and colleagues described an inducible MyD88/CD40 protein switch coupled with ectopic IL-15 that augmented antitumor activity through enhanced CAR-NK expansion targeting CD123 or BCMA [94]. In a recently published study, CAR-NK cell function in vivo and in xenograft models was boosted with a fourth-generation CAR-IL15 construct coupled with a CRISPR/Cas9-mediated knockout of CIS [95]. There are ongoing trials to incorporate IL-15 in CAR-NK cells that target B cell tumors (NCT03056339 and NCT04245722), and there are products in the manufacturing pipeline (FT596, TAK-007, NKX-101, etc.) [96].

The source of NK cells is another problem in manufacturing. Peripheral blood (PB) is the most commonly used source of mature NK cells, which strongly express activating receptors such as CD16, NKp44 and NKp46 as well as KIRs. PB-NK cells can be conveniently collected in a closed system after apheresis. However, isolating large numbers of PB-NK cells is a problem since only 5–15% of circulating blood lymphocytes are NK cells, and the quantities are highly donor-dependent [38]. Long expansion periods shorten telomeres and reduce cytotoxicity, but these challenges can be overcome by cytokine support and feeder cells (e.g., K562-mbIL15-41BBL) [97]. In clinical-scale products, NK cells derived from umblical cord blood (UCB) appear to be convenient in terms of lower risk of GVHD, low risk of viral transmission from donor to recipient, high availability of UCB units, less compelling HLA matching criteria and high proliferation capacity [72]. Although UCB-derived NK cells have immature phenotype and express lower levels of KIRs and granzyme B in comparison to PB-NK cells, the effector function can be improved with cytokines [98]. NK cell lines such as lymphoma-derived NK-92 are widely studied sources because of good proliferation rates and easy in vitro expansion procedures with the addition of recombinant IL-2. In most cell lines, NK cells lack CD16 expression or important activating receptors like natural cytotoxicity receptors (NCRs) [99,100]. NK cell lines need further investigation because they are unsafe and have limited efficacy because of the requirement to irradiate immortalized cell lines prior to infusion to prevent proliferation in patients [101]. Tang and colleagues applied CAR-NK-92 cells to treat less aggressive hematological malignancy and multiple myeloma and included suicide genes as a safety measure [81]. PB- and UCB-derived NK cells are donor-dependent and heterogenous which makes stem cell-derived NK cell products attractive, which can be generated from accessible sources such as fibroblasts or peripheral blood for “off-the shelf” use. Induced pluripotent stem cells (iPSCs) have shown to be equally as effective as PB-NK and NK-92 cells, along with not needing irradiation and being capable of ADCC [6]. Strategies to shorten and streamline the manufacturing process are evolving since a serum-, stromal- and feeder-free method was recently demonstrated to produce clinical-scale iPSC NK cells in 5 weeks [102]. 

Cryopreserving NK and CAR-NK cells is crucial in human trials because of the heterogeneity of NK cell donors. NK cells are sensitive to the freeze-thaw process; the viability and function of PB-NK cells are significantly decreased after thawing [17,103]. Conversely, UCB-derived NK cells and NK-92 cells recover well after cryopreservation [104]. The activity of frozen NK cells can be partially recovered by adding IL-2 [105]. A DMSO-free freezing medium composed of poly-lysine, dextran and ectoine improves post-thaw cytotoxicity [106]. Nanoparticle-mediated intracellular protection was shown to avoid cryoinjury [107].

Genetic modifications using viral vectors, transposons and CRISPR-Cas9 system are alternative strategies to improve cytotoxicity, persistence, tumor specificity and expansion of NK cells. Tumor recognition of NK cells can be enhanced by the fusion of full-length NKG2D protein with CD3ς as a combination of the concept of CAR design [108]. CRISPR/Cas9-mediated transcriptional activation of MICA triggered the NK cell anti-tumor responses (ADCC can be mediated by genetic modifications [109]). In another study, CRISPR/Cas9-mediated targeting of checkpoint molecule *Cish* gene improved the cytotoxicity towards B lymphoma cell line in vitro [110]. Notably, CD 16 undergoes cleavage by ADAM17 protease and is shed from the membrane following NK cell activation. In order to prevent this issue, a high-affinity non-cleavable version of the CD16a receptor was transduced into iPSCs, the CRISPR/Cas9 method was used to delete the ADAM17 gene or iPSCs were transduced with a recombinant Fcγ receptor [111,112,113]. 

Technical problems related to gene delivery into NK cells have hindered the progress in generating CAR-NK cells. Transfection with retrovirus may promote insertional mutations, but lentivirus transduction is unsuccessful for peripheral blood-derived NK cells due to NK cell resistance to viral transduction [114]. Mueller and colleagues demonstrated that with RD114 alpha-retroviruses, transduction efficiency was three times higher than for VSV-G lentiviruses up to day 7 following transduction [115]. To improve the safety of retroviral transfection in CAR-NK cell therapy, integrating suicide genes into CAR constructs could be an alternative strategy [81]. Lentiviral transduction efficiency improved using rosuvastatin [116] and BX795, an inhibitor or the TBK1/IKKε complex [117]. Recently, transduction efficacy has been shown to increase when baboon envelope pseudotyped lentivector was used to overcome the low binding capacity of the VSV-G lentivirus envelope glycoprotein to the low-density lipoprotein receptor (LDL-R) [118]. Cytokines and K562 feeder cells with membrane-bound IL-21/4-1BBL had a booster effect on not only expansion but also lentiviral and retroviral transduction of NK cells [119]. Non-viral transfection strategies such as electroporation seem like feasible options. Although expression is transient, achieving 95% of transfection efficiency is possible by using high-purity mRNA instead of cDNA in a plasmid. Some companies have already adapted electroporation in closed GMP-compliant production systems. Sleeping beauty transposon and CRISPR/Cas9-specific locus knock-in strategies need to be explored further for enhanced transduction and longer persistence in vivo [5].

## 6. Concluding Remarks

In conclusion, adoptive treatment with NK cells is a good candidate for combating hematological malignancies. It is a potential off-the-shelf product with broad clinical applications because there is no need for prior sensitization, the preparation process is easy and the product is reliable in avoiding adverse events. More convenient GMP-grade quality control systems and cryopreserved master cell banks need to be optimized for large-scale manufacturing. NK cell banking will come to the fore with more clinical data provided. Despite these advantages, a relevant percentage of patients are still non-responders to this treatment approach. For this reason, the discoveries of NK cell activation and function, improved cell expansion techniques, redirection of NK cells with chimeric antigen receptors (CARs), CRISPR/Cas9 gene editing, enhanced viral transduction and electroporation, clinical trials using autologous and allogeneic NK cells with or without novel therapeutic agents such as checkpoint inhibitors, mAbs and drugs effective in the tumor microenvironment will provide new perspectives for NK/CAR-NK cell-based immunotherapy.

## Figures and Tables

**Table 1:** Clinical trials with administration of autologous and allogeneic NK cells (aGVHD: Acute Graft versus Host Disease, AML: Acute Myeloid Leukemia, cGVHD: Chronic Graft versus Host Disease, CR: Complete Response, CRS: Cytokine Release Syndrome, CML: Chronic Myeloid Leukemia, Cy: Cyclophosphamide, DLI: Donor Lymphocyte Infusion, Flu: Fludarabine, F/U: Follow-up, GVHD: Graft versus Host Disease, iPSC: Induced Pluripotent Stem Cells, MDS: Myelodysplastic Syndrome, N/A: Not Applicable, NHL: Non-Hodgkin Lymphoma, ORR: Overall Response Rate, PBMC: Peripheral Blood Mononuclear Cells, R: Rituximab, SD: Stable Disease, UCB: Umbilical Cord Blood, * Posttransplant application). The numbers in the first column represent the number of patients.

Patients	Donor/NK Cell Source	NK Cell Expansion Method	Conditioning Regimen Prior to NK Infusion	Adverse Event/Toxicity	Response	Reference
4 Follicular Lymphoma, 5 Diffuse Large B Cell Lymphoma	Autologous/PBMC	IL-2 and IL-15 stimulation	None	None	CR in 7/9, median F/U: 44 months	[34]
9 AML	Allogeneic/PBMC	IL2, IL-12, IL-15, and IL-18 stimulation, CD3 depletion, CD56-positive selection	Flu + Cy	N/A	ORR 55%, CR 45%	[35]
4 AML, 1 CML	Haploidentical/PBMC	CD3 depletion, CD56 enrichment	None *	None	2/5 patients donor chimerism	[36]
19 AML	Haploidentical/PBMC	CD3 depletion, IL-2 stimulation	Flu + Cy	Pleural effusion in 1 patient	CR in 5/19	[14]
10 AML	Haploidentical/PBMC	CD3-depletion, CD56-enrichment, IL-2 stimulation	Flu + Cy	None	CR 100%	[37]
41 hematological malignancies	Haploidentical/PBMC	CD3-depletion, IL-15, IL-21 stimulation	None *	None	Significant reduction of leukemia progression 46% vs. 74% (historical cohort)	[38]
29 lymphoma	Autologous/PBMC	Ex vivo IL-2 stimulation	None	None	No change in outcome compared to historical controls	[33]
41 AML	Haploidentical/PBMC	CD3-depletion, IL-15, IL-21 and hydrocortisone stimulation	None *	Grade 2 to 4 aGVHD 28%, cGVHD 30%,fever 73%	CR 57%, 3-year leukemia progression 75%	[39]
6 B cell NHL	Allogeneic/PBMC	CD3-depletion, IL-2 stimulation	Flu + Cy + R	None	4/6 clinical response	[40]
7 AML	“Off-the-shelf”/NK-92	IL2 stimulation	None	None	1 blast reduction, 2 SD	[41]
26 AML	Haploidentical/PBMC	CD19 and CD3 depletion, rhIL15 stimulation	Flu + Cy	CRS in 56% of patients, neurologic toxicity in 5/9 patients	CR: 40%	[42]
8 AML, 5 CML	Haploidentical/PBMC	CD3-depletion K562 Clone9.mbIL21 feeder cells	None *	aGVHD grade 1–2 54%	CR: 11/13 median F/U: 14.7 months	[43]
9 AML	“Off-the-shelf“/iPSC	IL2 stimulation	Flu + Cy	3 patients Grade 3 febrile neutropenia	4/9 CR	[44]
11 B cell NHL	“Off-the-shelf“/iPSC	IL 2 stimulation	Flu + Cy	None	8/11 had objective response, CR median F/U: 5.2 months	[45]
3 AML	“Off-the-shelf“/iPSC	IL2 stimulation	Flu + Cy	None	1/3 CR	[44]
14 B cell NHL	“Off-the-shelf“/iPSC	IL2 stimulation	Flu + Cy + R	None	10/14 patients achieved objective response, 7 CR	[45]
10 AML	Allogeneic/UCB	CD34^+^ selection	Flu + Cy	None	4/10 disease free	[46]
12 MM	Allogeneic/UCB	CD3 depletion, K562-9.mbIL21, IL-2 stimulation	Lenalidomide/melphalan	None	10 patients achieved at least VGPR, Median F/U 21 months	[47]

**Table 2:** Several preclinical studies of CAR-NK cells in hematological malignancies.

Target	Tumor Type	NK Cell Source	Structure of CAR Constructs	References
CD19	B-cell leukemia	NK-92 cell line	Anti CD19 scFv + CD3ζ	[74]
CD19	B-cell leukemia	Peripheral blood	Anti CD19 scFv + 41BB-CD3ζ	[70]
CD19	B-cell malignancies	NK-92	Anti-CD19 scFV + CD3ζ, CD28 + CD3ζ or CD13 + CD3ζ	[71]
CD19	B-cell malignancies	Cord blood	Anti-CD19 scFv + 4-1BB + CD3ζ + iCasp9 + IL-15	[72]
CD19	B-cell malignancies	Peripheral blood	Anti CD19 scFv + 41BB + CD28 + CD3ζ	[75]
CD20	B-cell malignancies	Peripheral blood	Anti CD19 scFv + 41BB-CD3ζ	[76]
CD20	Burkitt lymphoma	Peripheral blood	Anti CD19 scFv + 41BB-CD3ζ + IL15	[77]
CD138	Multiple myeloma	NK-92MI	Anti CD19 scFv + CD3ζ	[78]
CS-1	Multiple myeloma	NK-92	Anti CD19 scFv + CD28 + CD3ζ	[79]
CD5	T-cell malignancies	NK-92	Anti CD19 scFv + 41BB + CD28 + CD3ζ	[80]

**Table 3:** Human trials of CAR-NK cells for hematological malignancies listed at ClinicalTrials.Gov (iPS: induced pluripotent stem).

Antigen Target	Tumor	NK Cell Source	Structure of the CAR Construct	Phase of the Study	ClinicalTrials.Gov Identifier # (Number)
CD22	B lymphoma	Unknown	Anti-CD22 + CD244	I	NCT03692767
CD19	B lymphoma	NK-92	Anti-C19 + CD244	I	NCT03690310
CD19/CD22	B lymphoma	Unknown	Anti-CD19/22 + CD244	I	NCT03824964
CD19	B lymphoma	Unknown	Unknown	I	NCT04639739
CD19	B lymphoma	Unknown	Unknown	I	NCT04887012
BCMA	Multiple myeloma	NK-92	Anti-BCMA + CD8αTM-4-1BB-CD3ζ	I/II	NCT03940833
CD7	NK/T-cell lymphoma	Unknown	Unknown	I	NCT04264078
CD19	B-lymphoid malignancies	Cord blood NK cells	Anti-CD19 + CD28-CD3ζ	I/II	NCT03056339
CD33	Acute myeloid leukemia	NK-92	Anti-CD33 + CD28-4-1BB-CD3ζ	I/II	NCT02944162
CD7	T-cell leukemia/lymphoma	NK-92	Anti-CD7 + CD28-4-1BB-CD3ζ	I/II	NCT02742727
CD19	B-cell malignancies	NK-92	Anti-CD19 + CD28-4-1BB-CD3ζ	I/II	NCT02892695
CD19	B lymphoma	iPS-derived NK cells	Anti-CD19 + CD244	I	NCT03824951
CD19	B-cell leukemia	Peripheral blood	Anti-CD19 + CD8αΤΜ + 4-1ΒΒ + CD3ζ	I	NCT00995137

## References

[1] Tanaka J., Miller J.S. (2020). Recent progress in and challenges in cellular therapy using NK cells for hematological malignancies. Blood Rev..

[2] Fang F., Xiao W., Tian Z. (2018). Challenges of NK cell-based immunotherapy in the newera. Front. Med..

[3] Abel A.M., Yang C., Thakar M.S., Malarkannan S. (2018). Natural killer cells: Development maturation and clinical utilization. Front. Immunol..

[4] Moretta L., Pietra G., Vacca P., Pende D., Moretta F., Bertaina A., Mingari M.C., Locatelli F., Moretta A. (2016). Human NK cells: From surface receptors to clinical applications. Immunol. Lett..

[5] Gong Y., Klein Wolterink R.G.J., Wang J., Bos G.M.J., Germeraad W.T.V. (2021). Chimeric antigen receptor natural killer (CAR-NK) cell design and engineering for cancer therapy. J. Hematol. Oncol..

[6] Shankar K., Capitini C.M., Saha K. (2020). Genome engineering of induced pluripotent stem cells to manufacture natural killer cell therapies. Stem. Cell Res. Ther..

[7] Davis Z.B., Vallera D.A., Miller J.S., Felices M. (2017). Natural killer cells unleashed: Checkpoint receptor blockade and BiKE/TriKE utilization in NK-mediated antitumor immunotherapy. Semin. Immunol..

[8] Mehta R.S., Rezvani K. (2018). Chimeric antigen receptor expressing natural killer cells forthe immunotherapy of cancer. Front. Immunol..

[9] Eberl G., Colonna M., Di Santo J.P., McKenzie A.N. (2015). Innate lymphoid cells. Innate lymphoid cells: A new paradigm in immunology. Science.

[10] Montaldo E., Vacca P., Moretta L., Mingari M.C. (2014). Development of human natural killer cells and other innate lymphoid cells. Semin. Immunol..

[11] Sivori S., Vacca P., Del Zotto G., Munari E., Mingari M.C., Moretta L. (2019). Human, NK cells: Surface receptors, inhibitory checkpoints, and translational applications. Cell Mol. Immunol..

[12] Sivori S., Pende D., Quatrini L., Pietra G., Della Chiesa M., Vacca P., Tumino N., Moretta F., Mingari M.C., Locatelli F. (2020). NK cells and ILCs in tumor immunotherapy. Mol. Aspects Med..

[13] Vivier E., Raulet D.H., Moretta A., Caligiuri M.A., Zitvogel L., Lanier L.L., Yokoyama W.M., Ugolini S. (2011). Innate or adaptive immunity? The example of natural killer cells. Science.

[14] Miller J.S., Soignier Y. (2005). Successful adoptive transfer and in vivo expansion of human haploidentical NK cells in patients with cancer. Blood.

[15] Cooper M.A., Bush J.E., Fehniger T.A., VanDeusen J.B., Waite R.E., Liu Y., Aguila H.L., Caligiuri M.A. (2002). In vivo evidence for a dependence on interleukin 15 for survival of natural killer cells. Blood.

[16] Miller J., Rooney C., Curtsinger J., McElmurry R., McCullar V., Verneris M.R., Lapteva N., McKenna D., Wagner J.E., Blazar B.R. (2014). Expansion and homing of adoptively transferred human natural killer cells in immunodeficient mice varies with product preparation and in vivo cytokine administration: Implications for clinical therapy. Biol. Blood Marrow. Transplant..

[17] Fehniger T.A., Cooper M.A. (2016). Harnessing NK cell memory for cancer immunotherapy. Trends Immunol..

[18] Romee R., Schneider S.E., Leong J.W., Chase J.M., Keppel C.R., Sullivan R.P., Cooper M.A., Fehniger T.A. (2012). Cytokine activation induces human memory-like NK cells. Blood.

[19] Ghofrani J., Lucar O., Dugan H., Reeves R.K., Jost S. (2019). Semaphorin 7A modulates cytokine- induced memory-like responses by human natural killer cells. Eur. J. Immunol..

[20] Lamb M.G., Rangarajan H.G., Tullius B.P., Lee D.A. (2021). Natural killer cell therapy for hematologic malignancies: Successes, challenges, and the future. Stem. Cell Res. Ther..

[21] Khaznadar Z., Boissel N., Agaugue S., Henry G., Cheok M., Vignon M., Geromin D., Cayuela J.M., Castaigne S., Pautas C. (2015). Defective, NK cells in acute myeloid leukemia patients at diagnosis are associated with blast transcriptional signatures of immune evasion. J. Immunol..

[22] Stringaris K., Sekine T., Khoder A., Alsuliman A., Razzaghi B., Sargeant R., Pavlu J., Brisley G., de Lavallade H., Sarvaria A. (2014). Leukemia-induced phenotypic and functional defects in natural killer cells predict failure to achieve remission in acute myeloid leukemia. Haematologica.

[23] Kearney C.J., Ramsbottom K.M., Voskoboinik I., Darcy P.K., Oliaro J. (2016). Loss of DNAM-1 ligand expression by acute myeloid leukemia cells renders them resistant to NK cell killing. Oncoimmunology.

[24] Scoville S.D., Nalin A.P., Chen L., Chen L., Zhang M.H., McConnell K., Beceiro Casas S., Ernst G., Traboulsi A.A., Hashi N. (2018). Human AML activates the aryl hydrocarbon receptor pathway to impair NK cell development and function. Blood.

[25] Clayton A., Mitchell J.P., Court J., Linnane S., Mason M.D., Tabi Z. (2008). Human tumor-derived exosomes down-modulate NKG2D expression. J. Immunol..

[26] Reiners K.S., Topolar D., Henke A., Simhadri V.R., Kessler J., Sauer M., Bessler M., Hansen H.P., Tawadros S., Herling M. (2013). Soluble ligands for NK cell receptors promote evasion of chronic lymphocytic leukemia cells from NK cell anti-tumor activity. Blood.

[27] Manso B.A., Zhang H., Mikkelson M.G., Gwin K.A., Secreto C.R., Ding W., Parikh S.A., Kay N.E., Medina K.L. (2019). Bone marrow hematopoietic dysfunction in untreated chronic lymphocytic leukemia patients. Leukemia.

[28] Balsamo M., Manzini C., Pietra G., Raggi F., Blengio F., Mingari M.C., Varesio L., Moretta L., Bosco M.C., Vitale M. (2013). Hypoxia downregulates the expression of activating receptors involved in NK-cell-mediated target cell killing without affecting ADCC. Eur. J. Immunol..

[29] Vigano S., Alatzoglou D., Irving M., Ménétrier-Caux C., Caux C., Romero P., Coukos G. (2019). Targeting, adenosine in cancer, immunotherapy to enhance, T-cell, function. Front. Immunol..

[30] Barsoum I.B., Hamilton T.K., Li X., Cotechini T., Miles E.A., Siemens D.R., Graham C.H. (2011). Hypoxia induces escape from innate immunity in cancer cells via increased expression of ADAM10: Role of nitric oxide. Cancer Res..

[31] Kikushige Y., Miyamoto T., Yuda J., Jabbarzadeh-Tabrizi S., Shima T., Takayanagi S., Niiro H., Yurino A., Miyawaki K., Takenaka K. (2015). A TIM-3/Gal-9 autocrine stimulatory loop drives self-renewal of human myeloid leukemia stem cells and leukemic progression. Cell Stem Cell.

[32] Vari F., Arpon D., Keane C., Hertzberg M.S., Talaulikar D., Jain S., Cui Q., Han E., Tobin J., Bird R. (2018). Immune evasion via PD-1/PD-L1 on NK cells and monocyte/macrophages is more prominent in Hodgkin lymphoma than DLBCL. Blood.

[33] Burns L.J., Weisdorf D.J., DeFor T.E., Vesole D.H., Repka T.L., Blazar B.R., Burger S.R., Panoskaltsis-Mortari A., Keever-Taylor C.A., Zhang M.J. (2003). IL-2-based immunotherapy after autologous transplantation for lymphoma and breast cancer induces immune activation and cytokine release: A phase I/II trial. Bone Marrow Transplant..

[34] Tanaka J., Tanaka N., Wang Y.H., Mitsuhashi K., Ryuzaki M., Iizuka Y., Watanabe A., Ishiyama M., Shinohara A., Kazama H. (2020). Phase, I study of cellular therapy using ex vivo expanded natural killer cells from autologous peripheral blood mononuclear cells combined with rituximab-containing chemotherapy for relapsed CD20-positive malignant lymphoma patients. Haematologica.

[35] Romee R., Rosario M., Berrien-Elliott M.M., Wagner J.A., Jewell B.A., Schappe T., Leong J.W., Abdel-Latif S., Schneider S.E., Willey S. (2016). Cytokine-induced memory-like natural killer cells exhibit enhanced responses against myeloid leukemia. Sci. Transl. Med..

[36] Passweg J.R., Tichelli A., Meyer-Monard S., Heim D., Stern M., Kühne T., Favre G., Gratwohl A. (2004). Purified donor NK-lymphocyte infusion to consolidate engraftment after haploidentical stem cell transplantation. Leukemia.

[37] Rubnitz J.E., Inaba H., Ribeiro R.C., Pounds S., Rooney B., Bell T., Pui C.H., Leung W. (2010). NKAML: A pilot study to determine the safety and feasibility of haploidentical natural killer cell transplantation in childhood acute myeloid leukemia. J. Clin. Oncol..

[38] Yoon S.R., Lee Y.S., Yang S.H., Ahn K.H., Lee J.-H., Lee J.-H., Kim D.Y., Kang Y.A., Jeon M., Seol M. (2010). Generation of donor natural killer cells fromCD34 progenitor cells and subsequent infusion after HLA-mismatched allogeneic hematopoietic cell transplantation: A feasibility study. Bone Marrow Transplant..

[39] Choi I., Yoon S.R., Park S.Y., Kim H., Jung S.J., Jang Y.J., Kang M., Yeom Y.I., Lee J.L., Kim D.Y. (2014). Donor-derived natural killer cells infused after human leukocyte antigen-haploidentical hematopoietic cell transplantation: A dose-escalation study. Biol. Blood Marrow Transplant..

[40] Bachanova V., Burns L.J., McKenna D.H., Curtsinger J., Panoskaltsis-Mortari A., Lindgren B.R., Cooley S., Weisdorf D., Miller J.S. (2010). Allogeneic natural killer cells for refractory lymphoma. Cancer Immunol. Immunother..

[41] Boyiadzis M., Agha M., Redner R.L., Sehgal A., Im A., Hou J.Z., Farah R., Dorritie K.A., Raptis A., Lim S.H. (2017). Phase 1 clinical trial of adoptive immunotherapy using “off-the-shelf” activated natural killer cells in patients with refractory and relapsed acute myeloid leukemia. Cytotherapy.

[42] Cooley S., He F., Bachanova V., Vercellotti G.M., DeFor T.E., Curtsinger J.M., Robertson P., Grzywacz B., Conlon K.C., Waldmann T.A. (2019). First-in-human trial of rhIL-15 and haploidentical natural killer cell therapy for advanced acute myeloid leukemia. Blood Adv..

[43] Ciurea S.O., Schafer J.R., Bassett R., Denman C.J., Cao K., Willis D., Rondon G., Chen J., Soebbing D., Kaur I. (2017). Phase 1 clinical trial using mbIL21 ex vivo-expanded donor-derived NK cells after haploidentical transplantation. Blood.

[44] Fate, Therapeutics Announces, Encouraging Interim, Phase 1 Data for iPSC-Derived NK Cell, Programs in Relapsed/Refractory, Acute Myeloid, Leukemia. https://ir.fatetherapeutics.com/news-releases/news-release-details/fate-therapeutics-announces-encouraging-interim-phase-1-data.

[45] Fate, Therapeutics Announces, Positive Interim, Clinical Data from its FT596 and FT516 Off-the-shelf, iPSC-Derived NK Cell, Programs for B-cell Lymphoma. https://ir.fatetherapeutics.com/news-releases/news-release-details/fate-therapeutics-announces-positive-interim-clinical-data-its.

[46] Dolstra H., Roeven M.W.H., Spanholtz J., Hangalapura B.N., Tordoir M., Maas F., Leenders M., Bohme F., Kok N., Trilsbeek C. (2017). Successful, transfer of umbilical, cord blood, CD34^+^ hematopoietic, stem and progenitor-derived NK cells in older, acute myeloid, leukemia patients. Clin. Cancer Res..

[47] Shah N., Li L., McCarty J., Kaur I., Yvon E., Shaim H., Muftuoglu M., Liu E., Orlowski R.Z., Cooper L. (2017). Phase I study of cord blood-derived natural killer cells combined with autologous stem cell transplantation in multiple myeloma. Br. J. Haematol..

[48] Deng X., Terunuma H., Nieda M., Xiao W., Nicol A. (2012). Synergistic cytotoxicity of ex vivo expanded natural killer cells in combination with monoclonal antibody drugs against cancer cells. Int. Immunopharmacol..

[49] Wang W., Erbe A.K., Alderson K.A., Phillips E., Gallenberger M., Gan J., Campana D., Hank J.A., Sondel P.M. (2016). Human NK cells maintain licensing status and are subject to killer immunoglobulin-like receptor (KIR) and KIR-ligand inhibition following ex vivo expansion. Cancer Immunol. Immunother..

[50] Kohrt H.E., Thielens A., Marabelle A., Sagiv-Barfi I., Sola C., Chanuc F., Fuseri N., Bonnafous C., Czerwinski D., Rajapaksa A. (2014). Anti-KIR antibody enhancement of anti-lymphoma activity of natural killer cells as monotherapy and in combination with anti-CD20 antibodies. Blood.

[51] Vey N., Karlin L., Sadot-Lebouvier S., Broussais F., Berton-Rigaud D., Rey J., Charbonnier A., Marie D., Andre P., Paturel C. (2018). A phase 1 study of lirilumab (antibody against killer immunoglobulin-like receptor antibody KIR2D; IPH2102) in patients with solid tumors and hematologic malignancies. Oncotarget.

[52] Carlsten M., Korde N., Kotecha R., Reger R., Bor S., Kazandjian D., Landgren O., Childs R.W. (2016). Checkpoint inhibition of KIR2D with the monoclonal antibody IPH2101 induces contraction and hyporesponsiveness of NK cells in patients with myeloma. Clin. Cancer Res..

[53] Bagot M., Porcu P., Marie-Cardine A., Battistella M., William B.M., Vermeer M., Whittaker S., Rotolo F., Ram-Wolff C., Khodadoust M.S. (2019). IPH4102, a first-in-class anti-KIR3DL2 monoclonal antibody, in patients with relapsed or refractory cutaneous T-cell lymphoma: An international, first-in-human, open-label, phase 1 trial. Lancet Oncol..

[54] Wang Y., Zhang Y., Hughes T., Zhang J., Caligiuri M.A., Benson D.M., Yu J. (2018). Fratricide of NK cells in daratumumab therapy for multiple myeloma overcome by ex vivo-expanded autologous NK cells. Clin. Cancer Res..

[55] Gleason M.K., Ross J.A., Warlick E.D., Lund T.C., Verneris M.R., Wiernik A., Spellman S., Haagenson M.D., Lenvik A.J., Litzow M.R. (2014). CD16xCD33 bispecific killer cell engager (BiKE) activates NK cells against primary MDS and MDSC CD33+ targets. Blood.

[56] Felices M., Kodal B., Hinderlie P., Kaminski M.F., Cooley S., Weisdorf D.J., Vallera D.A., Miller J.S., Bachanova V. (2019). Novel, CD19-targeted TriKE restores NK cell function and proliferative capacity in CLL. Blood Adv..

[57] Sarhan D., Brandt L., Felices M., Guldevall K., Lenvik T., Hinderlie P., Curtsinger J., Warlick E., Spellman S.R., Blazar B.R. (2018). 161533 TriKE stimulates NK-cell function to overcome myeloid-derived suppressor cells in MDS. Blood Adv..

[58] Shah N.N., Baird K., Delbrook C.P., Fleisher T.A., Kohler M.E., Rampertaap S., Lemberg K., Hurley C.K., Kleiner D.E., Merchant M.S. (2015). Acute GVHD in patients receiving IL-15/4-1BBL activated NK cells following T-cell-depleted stem cell transplantation. Blood.

[59] Bachanova V., Cooley S., Defor T.E., Verneris M.R., Zhang B., McKenna D.H., Curtsinger J., Panoskaltsis-Mortari A., Lewis D., Hippen K. (2014). Clearance of acute myeloid leukemia by haploidentical natural killer cells is improved using IL-2 diphtheria toxin fusion protein. Blood.

[60] Shi J., Tricot G., Szmania S., Rosen N., Garg T.K., Malaviarachchi P.A., Moreno A., Dupont B., Hsu K.C., Baxter-Lowe L.A. (2008). Infusion of haplo-identical killer immunoglobulin-like receptor ligand mismatched NK cells for relapsed myeloma in the setting of autologous stem cell transplantation. Br. J. Haematol..

[61] Lee D.A., Denman C.J., Rondon G., Woodworth G., Chen J., Fisher T., Kaur I., Fernandez-Vina M., Cao K., Ciurea S. (2016). Haploidentical, natural killer, cells infused before allogeneic, stem cell, transplantation for myeloid, malignancies: A phase I trial. Biol. Blood Marrow Transplant..

[62] Fehniger T.A., Miller J.S., Stuart R.K., Cooley S., Salhotra A., Curtsinger J., Westervelt P., DiPersio J.F., Hillman T.M., Silver N. (2018). A phase 1 trial of CNDO-109-activated, natural killer, cells in patients with high-risk, acute myeloid, leukemia. Biol. Blood Marrow Transplant..

[63] Zhang L., Liu M., Yang S., Wang J., Feng X., Han Z. (2021). Natural killer cells: Of-the-shelf cytotherapy for cancer immunosurveillance. Am. J. Cancer Res..

[64] Maude S.L., Laetsch T.W., Buechner J., Rives S., Boyer M., Bittencourt H., Bader P., Verneris M.R., Stefanski H.E., Myers G.D. (2018). Tisagenlecleucel in children and young, adults with B-cell, lymphoblastic leukemia. N. Engl. J. Med..

[65] Park J.H., Rivière I., Gonen M., Wang X., Sénéchal B., Curran K.J., Sauter C., Wang Y., Santomasso B., Mead E. (2018). Long-term, follow-up of CD19 CAR therapy in acute, lymphoblastic leukemia. N. Engl. J. Med..

[66] Daher M., Rezvani K. (2018). Next generation natural killer cells for cancer immunotherapy: The promise of genetic engineering. Curr. Opin. Immunol..

[67] Morris M.A., Ley K. (2004). Trafficking of natural killer cells. Curr. Mol. Med..

[68] Vitale M., Cantoni C., Della Chiesa M., Ferlazzo G., Carlomagno S., Pende D., Falco M., Pessino A., Muccio L., De Maria A. (2019). An historical overview: The discovery of how, NK cells can kill enemies recruit defense troops and more. Front. Immunol..

[69] Nazimuddin F., Finklestein J.M., Gupta M., Kulikovskaya I., Ambrose D.E., Gill S., Lacey S.F., Zheng Z., Melenhorst J.J., Levine B.L. (2013). Long-term functional persistence B cell aplasia and anti-leukemia efficacy in refractory B cell malignancies following T cell immunotherapy using CAR-redirected T cells targeting CD19. Am. Soc. Hematol..

[70] Shimasaki N., Fujisaki H., Cho D., Masselli M., Lockey T., Eldridge P., Leung W., Campana D. (2012). A clinically adaptable method to enhance the cytotoxicity of natural killer cells against B-cell malignancies. Cytotherapy.

[71] Oelsner S., Friede M.E., Zhang C., Wagner J., Badura S., Bader P., Ullrich E., Ottmann O.G., Klingemann H., Tonn T. (2017). Continuously expanding CAR NK-92 cells display selective cytotoxicity against B-cell leukemia and lymphoma. Cytotherapy.

[72] Liu E., Tong Y., Dotti G., Shaim H., Savoldo B., Mukherjee M., Orange J., Wan X., Lu X., Reynolds A. (2018). Cord blood NK cells engineered to express IL-15 and a CD19-targeted CAR show long-term persistence and potent antitumor activity. Leukemia.

[73] Li Y., Hermanson D.L., Moriarity B.S., Kaufman D.S. (2018). Human iPSC-derived natural kille cells engineered with chimeric, antigen receptors enhance anti-tumor activity. Cell Stem Cell.

[74] Romanski A., Uherek C., Bug G., Seifried E., Klingemann H., Wels W.S., Ottmann O.G., Tonn T. (2016). CD19-CAR engineered NK-92 cells are sufficient to overcome NK cell resistance in B-cell malignancies. J. Cell Mol. Med..

[75] Suerth J.D., Morgan M.A., Kloess S., Heckl D., Neudörfl C., Falk C.S., Koehl U., Schambach A. (2016). Efficient generation of gene-modified human natural killer cells via alpharetroviral vectors. J. Mol. Med..

[76] Chu Y., Hochberg J., Yahr A., Ayello J., van de Ven C., Barth M., Czuczman M., Cairo M.S. (2015). Targeting, CD20+ Aggressive, B-cell non-hodgkin lymphoma by anti-CD20 CAR mRNA-modified expanded natural killer cells in vitro and in nsg mice. Cancer Immunol. Res..

[77] Chu Y., Yahr A., Huang B., Ayello J., Barth M., Cairo M.S. (2017). Romidepsin alone or in combination with anti-CD20 chimeric antigen receptor expanded natural killer cells targeting Burkitt lymphoma *in vitro* and in immunodeficient mice. Oncoimmunology.

[78] Jiang H., Zhang W., Shang P., Zhang H., Fu W., Ye F., Zeng T., Huang H., Zhang X., Sun W. (2014). Transfection of chimeric anti-CD138 gene enhances natural killer cell activation and killing of multiple myeloma cells. Mol. Oncol..

[79] Chu J., Deng Y., Benson D.M., He S., Hughes T., Zhang J., Peng Y., Mao H., Yi L., Ghoshal K. (2014). CS1-specific chimeric antigen receptor (CAR)-engineered natural killer cells enhance in vitro and in vivo antitumor activity against human multiple myeloma. Leukemia.

[80] Chen K.H., Wada M., Pinz K.G., Liu H., Lin K.W., Jares A., Firor A.E., Shuai X., Salman H., Golightly M. (2017). Preclinical targeting of aggressive T-cell malignancies using anti-CD5 chimeric antigen receptor. Leukemia.

[81] Tang X., Yang L., Li Z., Nalin A.P., Dai H., Xu T., Yin J., You F., Zhu M., Shen W. (2018). First-in-man clinical trial of CAR NK-92 cells: Safety test of CD33-CAR NK-92 cells in patients with relapsed and refractory acute myeloid leukemia. Am. J. Cancer Res..

[82] Liu E., Marin D., Banerjee P., Macapinlac H.A., Thompson P., Basar R., Nassif Kerbauy L., Overman B., Thall P., Kaplan M. (2020). Use of CAR-transduced natural killer cells in CD19-positive lymphoid tumors. N. Engl. J. Med..

[83] Li X., He C., Liu C., Ma J., Ma P., Cui H., Tao H., Gao B. (2015). Expansion of NK cells from PBMCs using immobilized 4-1BBL and interleukin-21. Int. J. Oncol..

[84] Denman C.J., Senyukov V.V., Somanchi S.S., Phatarpekar P.V., Kopp L.M., Johnson J.L., Singh H., Hurton L., Maiti S.N., Huls M.H. (2012). Membrane-bound IL-21 promotes sustained ex vivo proliferation of human natural killer cells. PLoS ONE.

[85] Fujisaki H., Kakuda H., Imai C., Mullighan C.G., Campana D. (2009). Replicative potential of human natural killer cells. Br. J. Haematol..

[86] Jiang B., Wu X., Li X.N., Yang X., Zhou Y., Yan H., Wei A.H., Yan W. (2014). Expansion of NK cells by engineered K562 cells co-expressing 4-1BBL and mMICA, combined with soluble IL-21. Cell Immunol..

[87] Phan M.T., Lee S.H., Kim S.K., Cho D. (2016). Expansion of NK cells using genetically engineered K562 feeder cells. Methods Mol. Biol..

[88] Fujisaki H., Kakuda H., Shimasaki N., Imai C., Ma J., Lockey T., Eldridge P., Leung W.H., Campana D. (2009). Expansion of highly cytotoxic human natural killer cells for cancer cell therapy. Cancer Res..

[89] Chanswangphuwana C., Allan D.S.J., Chakraborty M., Reger R.N., Childs R.W. (2021). Augmentation of NK cell proliferation and anti-tumor immunity by transgenic expression of receptors for EPO or TPO. Mol. Ther..

[90] Sutlu T., Stellan B., Gilljam M., Quezada H.C., Nahi H., Gahrton G., Alici E. (2010). Clinical-grade, large-scale, feeder-free expansion of highly active human natural killer cells for adoptive immunotherapy using an automated bioreactor. Cytotherapy.

[91] McKenna D.H., Sumstad D., Bostrom N., Kadidlo D.M., Fautsch S., McNearney S., Dewaard R., McGlave P.B., Weisdorf D.J., Wagner J.E. (2007). Good manufacturing practices production of natural killer cells for immunotherapy: A six-year single-institution experience. Transfusion.

[92] Fernández A., Navarro-Zapata A., Escudero A., Matamala N., Ruz-Caracuel B., Mirones I., Pernas A., Cobo M., Casado G., Lanzarot D. (2021). Optimizing the procedure to manufacture clinical-grade NK cells for adoptive immunotherapy. Cancers.

[93] Vacca P., Pietra G., Tumino N., Munari E., Mingari M.C., Moretta L. (2020). Exploiting human NK cells in tumor therapy. Front. Immunol..

[94] Wang X., Jasinski D.L., Medina J.L., Spencer D.M., Foster A.E., Bayle J.H. (2020). Inducible MyD88/CD40 synergizes with IL-15 to enhance antitumor efficacy of CAR-NK cells. Blood Adv..

[95] Daher M., Basar R., Gokdemir E., Baran N., Uprety N., Nunez Cortes A.K., Mendt M., Kerbauy L.N., Banerjee P.P., Shanley M. (2021). Targeting a cytokine checkpoint enhances the fitness of armored cord blood CAR-NK cells. Blood.

[96] Islam R., Pupovac A., Evtimov V., Boyd N., Shu R., Boyd R., Trounson A. (2021). Enhancing a natural killer: Modification of NK cells for cancer, immunotherapy. Cells.

[97] Lapteva N., Durett A.G., Sun J., Rollins L.A., Huye L.L., Fang J., Dandekar V., Mei Z., Jackson K., Vera J. (2012). Large-scale ex vivo expansion and characterization of natural killer cells for clinical applications. Cytotherapy.

[98] Luevano M., Daryouzeh M., Alnabhan R., Querol S., Khakoo S., Madrigal A., Saudemont A. (2012). The unique profile of cord blood natural killer cells balancesincomplete maturation and effective killing function upon activation. Hum. Immunol..

[99] Luevano M., Madrigal A., Saudemont A. (2012). Generation of natural killer cells from hematopoietic stem cells in vitro for immunotherapy. Cell Mol. Immunol..

[100] Maki G., Klingemann H.-G., Martinson J.A., Tam Y.K. (2001). Factors regulating the cytotoxic activity of the human natural killer cell line NK-92. J. Hematother. Stem Cell Res..

[101] Tonn T., Schwabe D., Klingemann H.G., Becker S., Esser R., Koehl U., Suttorp M., Seifried E., Ottmann O.G., Bug G. (2013). Treatment of patients with advanced cancer with the natural killer cell line NK-92. Cytotherapy.

[102] Zhu H., Kaufman D.S. (2019). An improved method to produce clinical-scale natural killer cells from human pluripotent stem cells. Methods Mol. Biol..

[103] Van Ostaijen-ten Dam M.M., Prins H.J., Boerman G.H., Vervat C., Pende D., Putter H., Lankester A., van Tol M.J., Zwaginga J.J., Schilham M.W. (2016). Preparation of cytokine-activated NK cells for use in adoptive cell therapy in cancer patients: Protocol optimization and therapeutic potential. J. Immunother..

[104] Mehta R.S., Shpall E.J., Rezvani K. (2016). Cord blood as a source of natural killer cells. Front. Med..

[105] Pasley S., Zylberberg C., Matosevic S. (2017). Natural killer-92 cells maintain cytotoxicactivity after long-term cryopreservation in novel DMSO-free media. Immunol. Lett..

[106] Domogala A., Madrigal J.A., Saudemont A. (2016). Cryopreservation has no effect on function of natural killer cells differentiated in vitro from umbilical cord blood CD34(+) cells. Cytotherapy.

[107] Yao X., Jovevski J.J., Todd M.F., Xu R., Li Y., Wang J., Matosevic S. (2020). Nanoparticle-mediated intracellular protection of natural killer cells avoids cryoinjury and retains potent antitumor functions. Adv. Sci..

[108] Chang Y.-H., Connolly J., Shimasaki N., Mimura K., Kono K., Campana D. (2013). A chimeric receptor with NKG2D specificity enhances natural killer cell activation and killing of tumor cells. Cancer Res..

[109] Sekiba K., Yamagami M., Otsuka M., Suzuki T., Kishikawa T., Ishibashi R., Ohno M., Sato M., Koike K. (2017). Transcriptional activation of the MICA gene with an engineered CRISPR-Cas9 system. Biochem. Biophys. Res. Commun..

[110] Rautela J., Surgenor E., Huntington D.N. (2018). Efficient genome editing of human natural killer cells by CRISPR RNP. bioRxiv.

[111] Jing Y., Ni Z., Wu J., Higgins L.A., Markowski T.W., Kaufman D.S., Walcheck B. (2015). Identification of an ADAM17 cleavage region in human CD16 (FcγRIII) and the engineering of a non-cleavable version of the receptor in NK cells. PLoS ONE.

[112] Blum R., Arumugam A., Wu J., Walcheck B., Kaufman D. (2016). Engineering human pluripotent stem cell-derived natural killer cells to prevent CD16a shedding for enhanced anti-tumor killing. Blood.

[113] Snyder K.M., Hullsiek R., Mishra H.K., Mendez D.C., Li Y., Rogich A., Kaufman D.S., Wu J., Walcheck B. (2018). Expression of a recombinant high affinity IgG fc receptor by engineered NK cells as a docking platform for therapeutic mAbs to target cancer cells. Front. Immunol..

[114] Imai C., Iwamoto S., Campana D. (2005). Genetic modification of primary natural killer cells overcomes inhibitory signals and induces specific killing of leukemic cells. Blood.

[115] Müller S., Bexte T., Gebel V., Kalensee F., Stolzenberg E., Hartmann J., Koehl U., Schambach A., Wels W.S., Modlich U. (2020). High cytotoxic efficiency of lentivirally and alpharetrovirally engineered CD19-specific chimeric antigen receptor natural, killer cells against acute lymphoblastic leukemia. Front. Immunol..

[116] Gong Y., Klein Wolterink R.G.J., Janssen I., Groot A.J., Bos G.M.J., Germeraad W.T.V. (2020). Rosuvastatin enhances VSV-G lentiviral transduction of NK cells via upregulation of the low-density lipoprotein receptor. Mol. Ther. Methods Clin. Dev..

[117] Sutlu T., Nyström S., Gilljam M., Stellan B., Applequist S.E., Alici E. (2012). Inhibition of intracellular antiviral defense mechanisms augments lentiviral transduction of human natural killer cells: Implications for gene therapy. Hum. Gene Ther..

[118] Colamartino A.B.L., Lemieux W., Bifsha P., Nicoletti S., Chakravarti N., Sanz J., Roméro H., Selleri S., Béland K., Guiot M. (2019). Efficient and robust NK-cell transduction with baboon envelope pseudotyped lentivector. Front. Immunol..

[119] Streltsova M.A., Barsov E., Erokhina S.A., Kovalenko E.I. (2017). Retroviral gene transfer into primary human NK cells activated by IL-2 and K562 feeder cells expressing membrane-bound IL-21. J. Immunol. Methods.

